# Effects of sleep insufficiency on spatial working memory in low-pressure and hypoxic environments

**DOI:** 10.1097/MD.0000000000030210

**Published:** 2022-09-02

**Authors:** Bingqi Li, Haotian Dong, Yanxiang Wang, Fangming Li, Xiaolei Gao, Hailin Ma, Lei Gao

**Affiliations:** a Plateau Brain Science Research Center, Tibet University/South China Normal University, Lhasa, China.

**Keywords:** insufficient sleep, late positive potential, low-pressure and low-oxygen environment, P2, spatial working memory, time–frequency analysis

## Abstract

This study aimed to investigate the effects of sleep insufficiency on spatial working memory in low-pressure and hypoxic environments.

**Methods:**

We selected 58 insufficient sleepers and 27 normal sleepers among the college students living in high-altitude areas for a long time to receive a spatial 2-back working memory task, while collecting behavioral and electroencephalograph data. We adopted an independent sample *t*-test and repeated measures analysis of variance to compare the differences in response time and accuracy, P2 and late positive potential components, and theta band energy values in the spatial working memory task between insufficient and normal sleepers.

**Results:**

We found no significant differences in response time and accuracy between the insufficient sleep group and the normal sleep group; however, the P2 peak value and the early theta band energy value were higher in the insufficient sleep group than in the normal sleep group.

**Conclusions:**

These results suggest that the spatial working memory ability of individuals with sleep insufficiency was weakened under low-pressure and low-oxygen environment.

## 1. Introduction

The geographical environment and climatic characteristics of plateaus have diverse effects on human physiology and psychology, such as headaches, insomnia, chest tightness, asthma, and fatigue.^[1]^ Sleep disorders are more common and prominent among all symptoms.^[2,3]^ People who live at high altitudes for extended lengths of time experience unstable breathing due to low blood oxygen levels and frequent awakenings at night, resulting in sleep insufficiency.^[4,5]^ Kabel et al^[6]^ studied the relationship between long-term high-altitude exposure and the incidence of sleep insufficiency in adolescents and adults, and found a strong association between long-term high-altitude exposure and increased incidence of sleep insufficiency. Using the Pittsburgh Sleep Quality Questionnaire, Gupta et al^[7]^ found that the incidence of sleep insufficiency was approximately twice as high in adults living in high-altitude areas as compared to those in low-altitude areas.

Studies related to sleep have shown that the impact of sleep insufficiency on neurobehavior extends from simple cognitive measurements to more complex judgments and decision-making.^[8,9]^ Working memory is an important component of cognitive neuroscience research^[10,11]^ and includes form-specific information storage systems comprising a phonological loop that processes verbal information, a visuospatial sketchpad for processing spatial information, and a central executive system consisting of 3 cognitive processes (inhibition, shifting, and updating).^[12]^ Peng^[13]^ in their study found that sleep insufficiency impaired the working memory ability of college students.

The N-back task is the main research paradigm used to study working memory. From the perspective of information processing, the N-back task includes 2 processes: the matching process and refresh and short-term memory processes.^[14,15]^ Previous event-related potential (ERP) studies using the N-back paradigm showed that the P2 component and late positive potential (LPP) component were the main electroencephalograph (EEG) components associated with spatial working memory.^[16,17]^ The P2 component of the prefrontal lobe reflects the allocation of attentional resources in the early coding stage of working memory. When more attentional resources are invested in the coding stage, a larger amplitude of the P2 component is induced.^[18]^ LPP is a late positive component that reflects the allocation of attention resources during the late extraction of matching and mismatching information, and is located in the parietal lobe and prefrontal lobes.^[19]^ Greater the resource consumption, the greater the amplitude of the LPP component.^[20]^ In addition, the theta band is the major band associated with working memory. Givens^[21]^ showed that the theta band is closely related to working memory. Raghavachari et al^[22]^ believed that the theta band is the “gate” to spatial working memory, and as long as the spatial working memory is processing, the theta band energy value will increase; hence, the greater the cognitive load, the greater the theta energy value.

Studies have shown that exposure to high altitudes can easily lead to sleep insufficiency,^[6]^ which can impair individual working memory.^[13]^ However, individuals may have different sleep performances and processing patterns of spatial working memory in low-pressure and hypoxic environments. Therefore, we decided to use the spatial 2-back paradigm and combined ERP technology with it to investigate the effects of sleep insufficiency on spatial working memory under low-pressure and low-oxygen environments. We hypothesized that the sleep insufficiency group would have longer response times and lower accuracy than the normal sleep group; the insufficient sleep group would have greater P2 and LPP amplitudes and greater theta energy values.

## 2. Methods

### 2.1. Participants

We distributed 1460 Chinese versions of the Pittsburgh Sleep Quality Index (PSQI) and demographic information questionnaires to undergraduates of Xizang University and received 1280 effective questionnaires with an effective recovery rate of 87.67%. As per their responses to the demographic information questionnaire, the participants born and brought up (at least 18 years old) in the low-altitude area (<1000 m) and lived in Lhasa, Tibet (3650 m) for >6 months were screened out. Finally, we randomly selected 58 with insufficient sleepers (19 males, PSQI score > 7, 10.810 ± 2.578) and 27 normal sleepers (9 males, PSQI total score ≤ 7, 3.780 ± 1.695). All participants were of Han ethnicity (aged 20.54 ± 1.35 years), with normal or corrected-to-normal vision, and were right-handed. This study was approved by the Ethics Committee of Xizang University, and all participants signed informed consent before the experiment.

### 2.2. Stimuli and procedure

**Figure 1.**
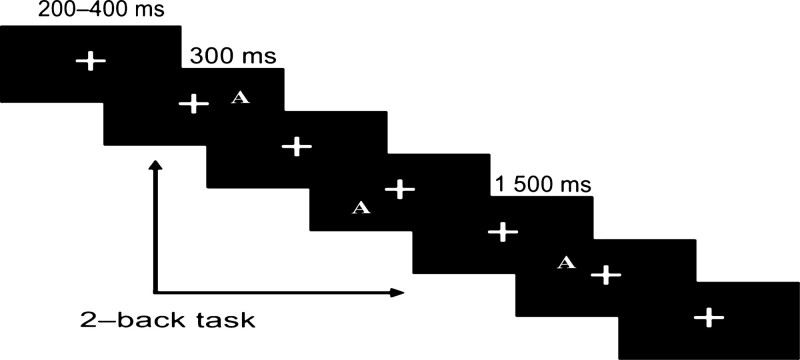
Stimuli and experiment procedure of this study. In this experiment, all trials began with a “+” fixation, which remained on the screen for a random time interval ranging from 200 to 400 ms, then a capital letter serving as the target stimulus was presented for 300 ms. If there was no response, the next trial started after 1500 ms. The stimuli were 12 capital letters (A to L) that would appear at 1 of 12 positions, each of which was at the tip of 1 of 6 equidistant radii of an imaginary circular array, 2 or 6 cm from the screen center. The participants were required to determine whether the positions of the letters presented at present were consistent with those displayed before the 2 trials.

The participants were seated in an electrically isolated, sound, and light-attenuated room, and viewed the computer monitor from a distance of 60 cm. In this experiment, all trials began with a “+” fixation, which remained on the screen for a random time interval ranging from 200 to 400 ms, then a capital letter serving as the target stimulus was presented for 300 ms. The stimuli were 12 capital letters (A to L) that would appear at 1 of 12 positions, each of which was at the tip of 1 of 6 equidistant radii of an imaginary circular array, 2 or 6 cm from the screen center. The stimulus occupied between 3° and 4.5° of the visual angle on either side of the visual midline. The participants were required to determine whether the positions of the letters presented at present were consistent with those displayed before the 2 trials. The experimental conditions were divided into left and right hand responses, and there were 4 blocks in total. Each block contained 74 trials, and the number of consistent and inconsistent trials were equal. In half of the trials, the participants were told to press the “C” key with their left index finger for a consistent stimulus and the “M” key with their right index finger for an inconsistent stimulus. In the other half of the trials, participants switched their hands. If there was no response, the next trial started after 1500 ms (Fig. 1).

### 2.3. EEG recording

Electroencephalography (EEG) data were recorded from 64 scalp sites (10/20 system) using Ag/AgCl electrodes mounted on an elastic cap (Neuroscan Inc., Charlotte, NC). The REF was used as the reference electrode and the horizontal electrooculogram was recorded by placing electrodes outside both eyes, and the vertical electrooculogram was recorded by placing electrodes above and below the left eye. The EEG and EOG data were continuously recorded at a sampling rate of 500 Hz by applying a bandwidth filter of 0.05 to 100 Hz. The electrode impedances were maintained at below 10 kΩ.

### 2.4. Collection and analysis of behavioral data

In this study, E-Prime 2.0 software was used to collect behavioral data, and SPSS software (version 23.0) was used to analyze the behavioral data. An independent sample *t* test was conducted to determine the response and accuracy of the insufficient sleep and normal sleep groups. The independent variables were insufficient sleep group and normal sleep group, and the dependent variables were response time and accuracy.

### 2.5. EEG data processing and statistical analysis

MATLAB (version R2018b; MathWorks, Inc, MA) invoked the EEGLAB14.1.1 toolkit^[23]^ to perform offline analysis of EEG data. The average of the left and right mastoids (M1 and M2) was used for offline re-reference. After continuous EEG recordings, the data were processed offline. Independent component analysis was performed using the EEGLAB toolbox, and ocular artifacts were manually removed. The ERP data were digitally low-pass filtered at 30 Hz and epoched into periods of 1200 ms, from 200 before to 1000 ms after the onset of each target stimulus. ERPLAB plug-in^[24]^ was used to delete trials with amplitudes exceeding ±100 μV, and only EEG data with correct responses were averaged. The following 9 sites were selected for LPP and P2 statistical analyses: anterior (FC1, FCZ, and FC2), middle (CP1, CPZ, and CP2), and posterior (PO3, POZ, and PO4). The time windows for the P2 and LPP components were 150–350 and 600–800 ms, respectively. Statistical software package (SPSS 23.0) was used to conduct a 2-factor repeated measurement analysis of variance of 2 (group: normal sleep group, insufficient sleep group) × 3 (electrode: anterior, middle, and posterior) for the average amplitude and peak value of the 2 components, to investigate whether there was a difference in EEG activity between the 2 groups. Finally, the Greenhouse–Geisser correction was used when the data violated the sphericity assumption.

### 2.6. Time–frequency analysis

MATLAB (version R2018b; MathWorks, Inc) invoked the FieldTrip toolkit for time–frequency analysis. The time–frequency analysis was performed by short-time Fourier transform and Hanning window analysis; the calculated time window was − 200 − 1000 ms, the frequency range was 0.1 to 30 Hz, the step size was 1 Hz, and the baseline correction of energy value is − 200 − 0. A total of 18 electrode points were selected for analysis: F1, FZ, F2, FC1, FCZ, and FC2 (front); C1, CZ, C2, CP1, CPZ, and CP2 (middle); P3, PZ, P4, PO3, POZ, and PO4 (rear). The time window of the time–frequency analysis is based on the time window of EEG data analysis, and the current research mainly focuses on the theta (4–8 Hz, 150–350/600–800 ms) frequency band. Statistical software package (SPSS 23.0) was used to conduct a 2-factor repeated measurement analysis of variance for the theta band energy values of 2 (group: normal sleep group, insufficient sleep group) × 3 (electrode: anterior, middle, and posterior), to investigate whether there was any difference in the theta band energy values between the 2 groups. Finally, the Greenhouse–Geisser correction was used when the data violated the sphericity assumption.

## 3. Results

### 3.1. Behavior results

Both the response time and the accuracy rate results showed that there was no significant difference between the insufficient sleep and normal sleep groups.

### 3.2. Time-domain results

**Figure 2.**
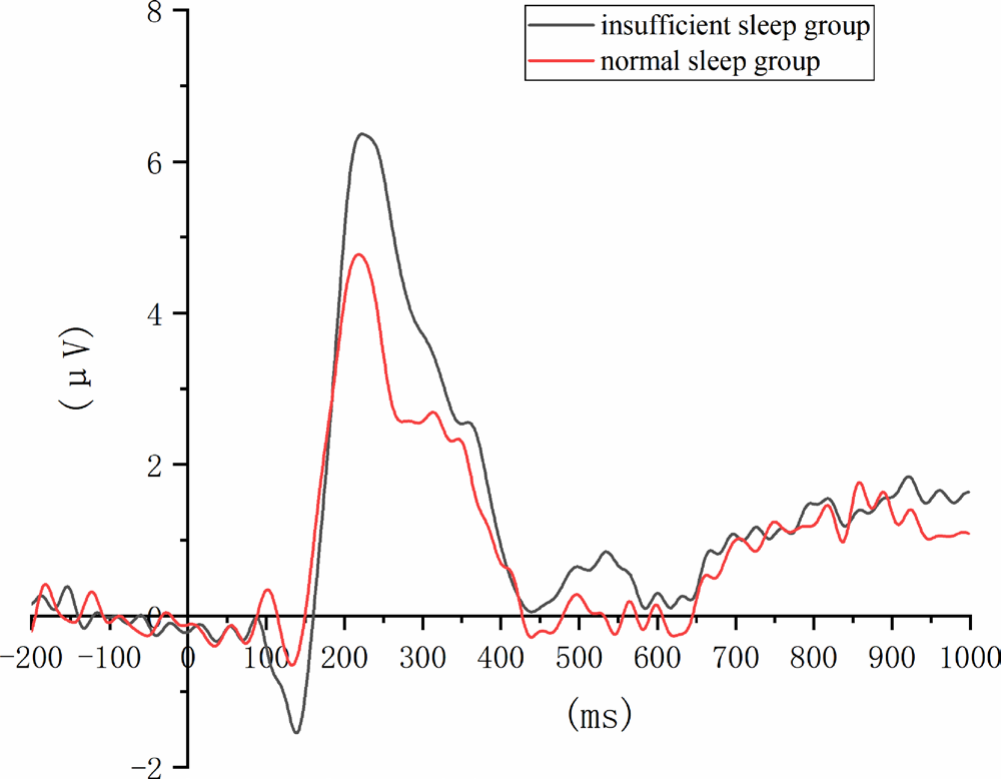
ERP waveform of the insufficient sleep and normal sleep groups. Contrast of ERP waveform between the 2 groups, in which the horizontal axis is time (ms) and the vertical axis is voltage value (μV). The 2 groups of participants are normal sleepers and insufficient sleepers, respectively. The time window for P2 is 150 to 350 ms, and that for LPP is 600 to 800 ms. The P2 component and LPP were the main EEG components associated with spatial working memory. As can be seen in the figure, the amplitude of both P2 and LPP in the insufficient sleep group is higher than that in the normal sleep group, indicating that the insufficient sleep group needs to consume more cognitive resources in spatial working memory to achieve the same behavioral results. EEG = electroencephalograph, ERP = event-related potential, LPP = late positive potential.

**Figure 3.**
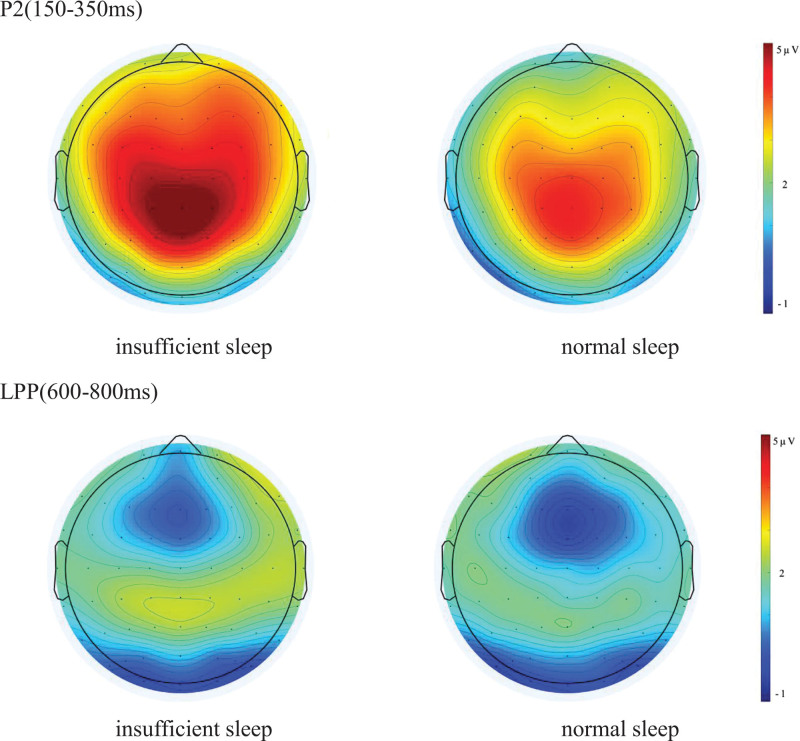
ERP topography of the insufficient sleep and normal sleep groups. Contrast of ERP topography between the 2 groups. The 2 groups of participants are normal sleepers and insufficient sleepers, respectively. As indicated by the ruler, the color change represents the difference in voltage amplitude. As shown in the figure, the voltage amplitude of the P2 (150–350 ms) component is higher in the insufficient sleep group, indicating that the insufficient sleep group consumes more cognitive resources than the normal sleep group; the voltage amplitude of LPP (600–350 ms) component in the insufficient sleep group was larger than that in the normal sleep group. It is possible that the insufficient sleep group tended to consume more attentional resources than the normal sleep group. ERP = event-related potential, LPP = late positive potential.

By analyzing the peak value of the P2 component, the main effect of the group was significant (*F* (1,83) = 5.274, *P* = .024, partial *η*^2^ = 0.060), indicating that the P2 peak in the insufficient sleep group was significantly higher than that in the normal sleep group. The main effect of the electrode site was also significant (*F* (2,166) = 28.530, *P* < .001, partial *η*^2^ = 0.256), which showed that the peak value of P2 in the anterior and middle regions was significantly higher than that in the posterior regions. However, the interaction between the groups and electrode positions was not significant (Table 1; Figs. 2 and 3).

**Table 1:** Time-domain results of the insufficient sleep and normal sleep groups (M ± SE)

Component	Group		*F*	*P*	Partial *η*^2^
	Insufficient sleep	Normal sleep			
P2 peak value (μV)	8.675 ± 0.413	6.993 ± 0.605	5.274	.024	0.060
P2 mean amplitude (μV)	4.271 ± 0.302	3.444 ± 0.443	2.377	.127	0.028
LPP mean amplitude (μV)	0.660 ± 0.341	0.543 ± 0.500	0.037	.848	0.000
Component	Group		*t*	*P*	
	Insufficient sleep	Normal sleep			
P2 peak value (μV) anterior	9.251 ± 4.369	7.565 ± 2.182	1.894	.062	
P2 peak value (μV) middle	10.011 ± 4.324	7.790 ± 2.266	2.508	.014	
P2 peak value (μV) posterior	6.762 ± 3.734	5.624 ± 2.377	1.451	.151	
P2 mean amplitude (μV) anterior	4.558 ± 3.196	3.616 ± 2.116	1.393	.167	
P2 mean amplitude (μV) middle	5.459 ± 3.062	4.394 ± 2.293	1.607	.112	
P2 mean amplitude (μV) posterior	2.797 ± 2.516	2.321 ± 2.027	0.860	.392	
LPP mean amplitude (μV) anterior	0.704 ± 3.434	0.489 ± 2.771	0.286	.776	
LPP mean amplitude (μV) middle	1.617 ± 3.092	1.235 ± 2.738	0.550	.584	
LPP mean amplitude (μV) posterior	−0.342 ± 2.421	−0.094 ± 2.119	−0.457	.649	

Anterior (FC1, FCZ, and FC2); middle (CP1, CPZ, and CP2); posterior (PO3, POZ, and PO4).

LPP = late positive potential, M = mean, SE = standard error.

Regarding the mean amplitude of the P2 component, the main effect of the group was not significant. The main effect of the electrode site was significant (*F* (2,166) = 34.137, *P* < .001, partial *η*^2^ = 0.291), with a larger P2 amplitude in the anterior and middle regions than in the posterior region. The interaction between the groups and electrode positions was not significant (Table 1; Figs. 2 and 3).

With respect to the mean amplitude of the LPP component, the main effect of the group was not significant; however, the main effect of the electrode site was significant (*F* (2,166) = 21.574, *P* < .001, partial *η*^2^ = 0.206), which showed that the average amplitude of the LPP in the front and middle regions was significantly larger than that in the posterior region. The interaction between the groups and electrode positions was not significant (Table 1; Figs. 2 and 3).

### 3.3. Time–frequency results

**Figure 4:**
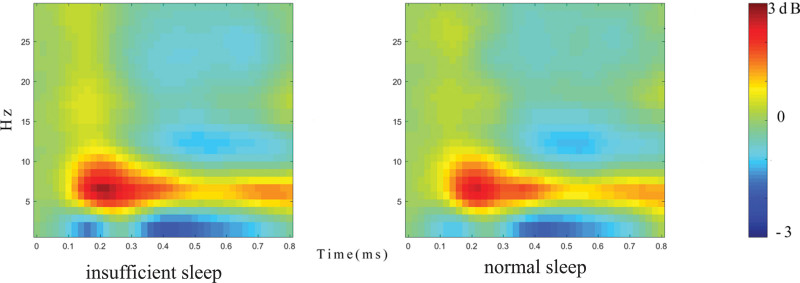
Frequency plots of the insufficient sleep and normal sleep groups. Contrast of frequency plots between the 2 groups. The 2 groups of participants are normal sleepers and insufficient sleepers respectively. The unit of the horizontal axis is time (ms) and the unit of the vertical axis is frequency (Hz). As shown in the ruler, the color change represents the power value and its unit is dB. In the early theta band (4–8 Hz, 150–350 ms), the early theta band power value of the insufficient sleep group was significantly higher than that of the normal sleep group, indicating that the insufficient sleep group consumed more attentional resources. In the late theta band (4–8 Hz, 600–800 ms). There was no difference in power values between the insufficient sleepers and normal sleepers.

Concerning the energy value of the early theta band (4–8 Hz, 150–350 ms), the main effect of the group was significant (*F* (1,83) = 4.047, *P* = .047, partial *η*^2^ = 0.046), with a higher energy value in the insufficient sleep group as compared to in the normal sleep group. The main effect of the electrode point was significant (*F* (2,166) = 10.077, *P* < .001, partial *η*^2^ = 0.108), indicating that the energy value of the central region was significantly larger than that of the theta band in the front region. The interaction between groups and electrode positions was not significant (Table 2; Fig. 4).

**Table 2:** Time–frequency results of the insufficient sleep and normal sleep groups (M ± SE).

Band	Group		*F*	*P*	Partial *η^2^*
	Insufficient sleep	Normal sleep			
Theta (4–8 Hz, 150–350 ms, dB)	1.989 ± 0.096	1.647 ± 0.141	4.047	.047	0.046
Theta (4–8 Hz, 600–800 ms, dB)	0.540 ± 0.103	0.451 ± 0.151	0.240	.625	0.003
Band	Group		*t*	*P*	
	Insufficient sleep	Normal sleep			
Theta (4–8 Hz,150–350 ms) front	1.768 ± 0.890	1.527 ± 0.571	1.288	.201	
Theta (4–8 Hz,150–350 ms) middle	2.074 ± 0.861	1.695 ± 0.616	2.051	.043	
Theta (4–8 Hz,150–350 ms) rear	2.125 ± 0.796	1.719 ± 0.826	2.166	.033	
Theta (4–8 Hz,600–800 ms) front	0.784 ± 0.973	0.734 ± 0.686	0.239	.812	
Theta (4–8 Hz,600–800 ms) middle	0.512 ± 0.891	0.399 ± 0.582	0.598	.551	
Theta (4–8 Hz,600–800 ms) rear	0.326 ± 0.890	0.220 ± 0.598	0.559	.578	

Front (F1, FZ, F2, FC1, FCZ, and FC2); middle (C1, CZ, C2, CP1, CPZ, and CP2); rear (P3, PZ, P4, PO3, POZ, and PO4).

M = mean, SE = standard error.

For the energy value of the late theta band (4–8 Hz, 600–800 ms), the main effect of group was not significant; however, the main effect of the electrode point was significant (*F* (2,166) = 32.703, *P* < .001, partial *η*^2^ = 0.283), with a greater energy value of the theta band in the anterior region than in the middle and posterior regions. The interaction between the groups and electrode positions was not significant (Table 2; Fig. 4).

## 4. Discussion

This study aimed to identify the effects of insufficient sleep on working memory under low-pressure and low-oxygen conditions. Working memory performance was measured using the N-back task combined with ERP recordings in the insufficient sleep and normal sleep groups. We found that there was no significant difference in response time and accuracy rate between the insufficient sleep and normal sleep groups in the behavioral results; in the ERP results, the peak value of the P2 component was significantly higher in the insufficient sleep group, and the early theta band energy value was significantly larger in the insufficient sleep group. Although we did not find a significant difference in the behavioral performance of spatial working memory between the 2 groups, significant differences were found in the ERP results, which may because ERP technical indicators are more sensitive than behavioral indicators.^[25]^There were no significant differences in behavioral outcomes between groups, and the disappearance of the effects of sleep insufficiency on behavior in low-pressure and hypoxic environments may be an adaptive mechanism supported by compensatory mechanisms.^[26]^

The frontal P2 component reflects a top-down process, matching sensory inputs to memory representations^[27,28]^; this is represented by an increase in the P2 amplitude in the early selection process.^[29]^ In our study, the P2 peak value in the insufficient sleep group was significantly higher than that in the normal sleep group, with a difference in the matching process of spatial working memory between the two groups. This finding suggests that the insufficient sleep group might have higher cognitive deficits in the matching process of spatial working memory.

According to previous studies, LPP is a late component of working memory and reflects the allocation of attentional resources during the matching stage of working memory. The more the attentional resources are consumed, the greater the amplitude of the LPP component.^[20,30]^ We found that although there was no significant difference in the mean amplitude of LPP between the insufficient sleep and normal sleep groups, the mean amplitude of LPP in the insufficient sleep group was larger than that in the normal sleep group. It is possible that the insufficient sleep group tended to consume more attentional resources than the normal sleep group.

Working memory is closely related to theta oscillations. Working memory tasks can induce significant theta oscillations, which increase with an increase in working memory load.^[22,31]^ In this study, we found that the early theta band energy value of the insufficient sleep group was significantly higher than that of the normal sleep group, indicating that the insufficient sleep group consumed more attentional resources and participated in a higher level of neural activity than the normal sleep group did.

Our study has some limitations. First, we only used questionnaires to screen subjects. Therefore, we are short of objective measures to verify participants’ sleep–wake times (e.g., actigraphy, polysomnography). Second, because college students with insufficient sleep were mostly women, we selected more female volunteers. Hence, we hesitate to extend our conclusions to men. In future research, we will use objective measures and increase male participants to study related issues; in addition, our procedure combines with fMRI to further explore differences in brain regions, which will facilitate a clearer picture of sleep insufficiency’s effects on working memory. Finally, we can also increase intervention methods and explore ways to solve the sleep insufficiency, which can help improve the sleep of college students.

In conclusion, our research showed that the spatial working memory of people with sleep insufficiency may be impaired under low-pressure and low-oxygen conditions. Compared with normal sleepers, the weakening of the spatial working memory of people with insufficient sleep involves a matching process and requires more attentional resources. Overall, this study provides valuable physiological evidence for understanding mechanisms underlying the effects of sleep insufficiency on spatial working memory.

## Acknowledgments

We acknowledge all participating teachers and students.

## Author contributions

**Project administration:** Yanxiang Wang, Fangming Li.

**Writing – original draft:** Bingqi Li, Haotian Dong.

**Writing – review & editing:** Xiaolei Gao, Hailin Ma, Lei Gao.
